# Clinical evaluation of a fully automated and high-throughput molecular testing system for detection of influenza virus

**DOI:** 10.1186/s12985-022-01916-w

**Published:** 2022-11-16

**Authors:** Kosuke Kosai, Norihito Kaku, Michiko Horie, Hina Kodama, Norihiko Akamatsu, Yusuke Narita, Yasushi Matsumoto, Tetsuro Matsushita, Yohei Mizuta, Koichi Izumikawa, Hiroshi Mukae, Katsunori Yanagihara

**Affiliations:** 1 Department of Laboratory Medicine, Nagasaki University Hospital, 1-7-1 Sakamoto, Nagasaki, 852-8501 Japan; 2 Department of Laboratory Medicine, Nagasaki University Graduate School of Biomedical Sciences, Nagasaki, Japan; 3 Medical Scientific Affairs Group, Roche Diagnostics K.K, Tokyo, Japan; 4 Narita Naika Clinic, Nagasaki, Japan; 5 Matsumoto Naika, Nagasaki, Japan; 6 Shinzato Medicare Group Shinzato Clinic, Nagasaki, Japan; 7 Menoto Hosiptal, Nagasaki, Japan; 8 Department of Infectious Diseases, Nagasaki University Graduate School of Biomedical Sciences, Nagasaki, Japan; 9 Department of Respiratory Medicine, Nagasaki University Graduate School of Biomedical Sciences, Nagasaki, Japan

**Keywords:** Automated molecular assay, Nucleic acid amplification test, Batch assay, Respiratory virus

## Abstract

**Introduction:**

We investigated the performance of the cobas® 6800 system and cobas SARS-CoV-2 & Influenza A/B, a fully automated molecular testing system for influenza viruses and severe acute respiratory syndrome coronavirus 2 (SARS-CoV-2). This enabled an assay in a batch of 96 samples in approximately 3 h.

**Methods:**

An assay was performed using the cobas SARS-CoV-2 & Influenza A/B on the cobas 6800 system for samples collected in four facilities between November 2019 and March 2020 in our previous study. The results were compared with those obtained using the reference methods.

**Results:**

Of the 127 samples analyzed, the cobas SARS-CoV-2 & Influenza A/B detected influenza A virus in 75 samples, of which 73 were positive using the reference methods. No false negative results were observed. The overall positive and negative percent agreement for influenza A virus detection were 100.0% and 96.3%, respectively. There were no positive results for the influenza B virus or SARS-CoV-2.

**Conclusion:**

The cobas 6800 system and cobas SARS-CoV-2 & Influenza A/B showed high accuracy for influenza A virus detection and can be useful for clinical laboratories, especially those that routinely assay many samples.

## Introduction

Influenza is a major respiratory pathogen that causes seasonal epidemics and outbreaks. Influenza is a self-limiting disease in most patients; however, some patients develop complications such as pneumonia, exacerbation of chronic obstructive pulmonary disease, myocarditis, and encephalopathy [1, 2].

Several methods are available for detecting influenza viruses, including antigen and nucleic acid amplification tests (NAATs). Although antigen testing is rapid and simple, its sensitivity is generally low [3]. NAAT is a reliable method with high sensitivity; however, conventional PCR is time consuming and labor intensive. Recently, automated molecular systems have been developed for the detection of influenza viruses. We recently reported the usefulness of a fully automated molecular point-of-care (POC) testing system, the cobas Influenza A/B & RSV on the cobas® Liat system, for influenza A virus detection. The assay is performed on a single sample within a short time (approximately 20 min) [4]. In contrast, the cobas® 6800 system, a fully automated molecular testing system, provides a batch assay for 96 samples in approximately 3 h. In this study, we evaluated the performance of the cobas 6800 system and cobas SARS-CoV-2 & Influenza A/B, which targeted severe acute respiratory syndrome coronavirus 2 (SARS-CoV-2) and influenza A and B viruses, focusing on the detection of influenza A virus.

## Methods

### Samples

In our previous study [4], we used nasopharyngeal swab samples collected from adult outpatients with one or more symptoms, such as fever (38 °C or an increase of 1 °C from normal body temperature), nasal discharge, nasal congestion, sore throat, cough, headache, chills, fatigue, joint pain, or muscle pain, at Menoto Hospital, Matsumoto Naika, Narita Naika Clinic, and Shinzato Medicare Group Shinzato Clinic between November 2019 and March 2020. Patients were excluded if they were treated with anti-influenza agents within 1 month prior to the outpatient visit. The remaining samples in the UTM (Copan Italia s.p.a.) from our previous study were stored at − 80 °C, and samples with sufficient residual volume for this study were randomly selected and used.

### Assay using the cobas SARS-CoV-2 & Influenza A/B on the cobas 6800 system

According to the manufacturer’s instructions, Roche Diagnostics K.K. performed the assay using cobas SARS-CoV-2 & Influenza A/B on a cobas 6800 system. Eight hundred microliters of the sample was transferred to a cobas omni secondary tube and loaded onto the assay machine. Sample preparation, reverse transcription of the target RNA to complementary DNA, and real-time multiplexed PCR were automatically performed on the system. The targets were genes of membrane proteins 1 and 2 for influenza A virus, genes of nonstructural and nuclear export proteins for influenza B virus, and open reading frame 1a/b and envelope protein genes for SARS-CoV-2. Positive, negative, and internal controls were used for each assay. The system displayed the validity of the test and detection results for each target. According to the manufacturer’s instructions, the limits of detection were 0.026–0.14, 0.017–0.053, and 0.0079–0.12 50% tissue culture infectious dose (TCID_50_)/mL for influenza A virus, influenza B virus, and SARS-CoV-2, respectively.

## Reference methods

The detection of influenza viruses was performed in our previous study [4] and the results were used as reference results. Briefly, samples were assayed using the cobas Influenza A/B & RSV on the cobas Liat system, as well as an automated immunochromatographic antigen test (digital immunoassay, DIA test). The concordant results between the two were considered true results. Samples with discrepant results for influenza A virus (positive in the molecular POC test but negative in the DIA test) were confirmed to be true-positive by RT-PCR. None of the samples tested negative in the molecular POC test but positive in the DIA test. Finally, all Liat Flu/RSV results for influenza A virus detection were considered true results.

Roche Diagnostics K.K. performed the assay for SARS-CoV-2 detection according to the Manual for the Detection of Pathogen 2019-nCoV Ver.2.9.1, published by the National Institute of Infectious Diseases in Japan. Briefly, RNA was extracted using the QIAamp Viral RNA Mini (QIAGEN), and one-step real-time reverse transcription-PCR was performed using the QuantiTect Prove RT-PCR kit (QIAGEN) on a LightCycler® 480 Instrument II (Roche Molecular Systems Inc.).

## Results

A total of 129 samples were assayed using the cobas SARS-CoV-2 & Influenza A/B. Two samples were excluded from the analysis due to invalid results. Comparisons between the cobas SARS-CoV-2 & Influenza A/B and the reference methods are presented in Table 1. Of the 127 samples tested, the cobas SARS-CoV-2 & Influenza A/B detected influenza A virus in 75 samples, of which 73 were positive using the reference methods. No samples tested negative on the cobas SARS-CoV-2 & Influenza A/B and positive with the reference methods. The overall positive and negative percent agreement (PPA and NPA) for influenza A virus detection were 100.0% and 96.3%, respectively. Influenza B virus was not detected by the cobas SARS-CoV-2 & Influenza A/B or by the reference methods.

**Table 1:** Comparison of detection results between cobas SARS-CoV-2 & Influenza A/B and the reference methods

Viruses	cobas+, RM+	cobas+, RM-	cobas-, RM+	cobas-, RM-	PPA	NPA
Influenza A virus	73	2	0	52	100.0 (95.1–100.0)	96.3 (87.3–99.5)
Influenza B virus	0	0	0	127	-	100.0 (97.1–100.0)
SARS-CoV-2	0	0	0	47	-	100.0 (92.5–100.0)

RM, reference method; PPA, positive percent agreement; NPA, negative percent agreement

SARS-CoV-2 was not detected by the cobas SARS-CoV-2 & Influenza A/B in 127 samples. Of these samples, 47 were analyzed using the reference method for SARS-CoV-2 detection, and the results were negative.

## Discussion

This study demonstrated that the cobas SARS-CoV-2 & Influenza A/B could accurately detect influenza A virus (PPA, 100.0%) using a simple procedure. Unfortunately, we could not evaluate its detection performance for SARS-CoV-2 because the samples used in this study were collected before the SARS-CoV-2 pandemic in our region.

Currently, many molecular POC tests for individual samples are available and provide rapid and accurate diagnosis [4–6], which might lead to the appropriate use of antiviral agents and the reduction of inappropriate antibiotic use. In contrast, the cobas 6800 system used in this study enables a batch assay and can simultaneously examine 96 samples (up to 384 samples in an 8-hour shift) [7]. This system can be useful in clinical laboratories that routinely assay a large number of samples.

Furthermore, if influenza epidemics and the coronavirus disease 2019 (COVID-19) pandemic occur simultaneously, it may be difficult to differentiate them based on only clinical presentation [8]. This implies the need for accurate diagnostic methods that can distinguish between influenza and COVID-19 and detect their co-infections.

## Conclusion

The cobas 6800 system and cobas SARS-CoV-2 & Influenza A/B showed high accuracy for influenza A virus detection and can be useful for clinical laboratories, especially those that assay many samples in daily practice.

## Data Availability

Not applicable.

## Abbreviations

NAAT: nucleic acid amplification test
POC: point-of-care
SARS-CoV-2: severe acute respiratory syndrome coronavirus
TCID_50_: 50% tissue culture infectious dose
PPA: positive percent agreement
NPA: negative percent agreement
